# Theory to Practice: Performance Preparation Models in Contemporary High-Level Sport Guided by an Ecological Dynamics Framework

**DOI:** 10.1186/s40798-020-00268-5

**Published:** 2020-08-14

**Authors:** Carl T. Woods, Ian McKeown, Mark O’Sullivan, Sam Robertson, Keith Davids

**Affiliations:** 1 Institute for Health and Sport, Victoria University, Melbourne, Victoria Australia; 2 Football Department, Port Adelaide Football Club, Adelaide, South Australia Australia; 3 Sport and Physical Activity Research Centre, Sheffield Hallam University, Sheffield, UK; 4 Research and Development Department, AIK Football, Stockholm, Sweden

**Keywords:** Praxis, Constraints-led approach, Self-regulation, Practice design, Association football, Australian football

## Abstract

A fundamental challenge for practitioners in high-level sporting environments concerns how to support athletes in adapting behaviours to solve emergent problems during competitive performance. Guided by an ecological dynamics framework, the design and integration of competitive performance preparation models that place athlete-environment interactions at the heart of the learning process may address this challenge. This ecological conceptualisation of performance preparation signifies a shift in a coach’s role; evolving from a consistent solution provider to a learning environment *designer* who fosters local athlete-environment interactions. However, despite the past decades of research within the ecological dynamics framework developing an evidence-based, theoretical conceptualisation of skill acquisition, expertise and talent development, an ongoing challenge resides within its practical integration into sporting environments. This article provides two case examples in which high-level sports organisations have utilised an ecological dynamics framework for performance preparation in Australian football and Association Football. A unique perspective is offered on experiences of professional sport organisations attempting to challenge traditional ideologies for athlete performance preparation by progressing the theoretical application of ecological dynamics. These case examples intend to promote the sharing of methodological ideas to improve athlete development, affording opportunities for practitioners and applied scientists to accept, reject or adapt the approaches presented here to suit their specific ecosystems.

## Key Points

- Ecological dynamics offers a theoretical framework to guide performance preparation in sport from high-performance to developmental environments.
- The use of ecological dynamics as a framework for performance preparation requires practitioners to view themselves as learning *designers* that promote athlete-environment interactions.
- The continued sharing of case exemplars within sport science could drive the methodological advancement of contemporary performance preparation models that offer practical use for sports practitioners.

## Introduction

“There is nothing so practical as a good theory”—Kurt Lewin (1951)

In high-level sport, practitioners are required to prepare athletes for the demands of present competitive performance environments, whilst concurrently developing athletes of the future. These tasks signify the implementation of practical support activity operating at two integrated, but different, timescales in the *micro-structure* of practice (undertaken hourly, daily, weekly and monthly) and at the *macro-structure* of talent development (over extended periods of many years) [1, 2]. The design and successful integration of performance preparation models capable of supporting athletes in regulating their performance behaviours in competition is, therefore, a priority in high-level sports organisations.

Athlete-environment interactions have been modelled as *complex adaptive systems* composed of many interacting parts or degrees of freedom, which need to be coordinated and continuously regulated in achieving task goals [1, 3]. Two main pathways have been proposed for learners to successfully satisfy the constraints of challenging performance environments: externally and internally driven [4]. Externally driven (re)organisation of degrees of freedom in athlete-environment systems develops from an external influence globally prescribing instructions and directions, for example, from a parent/caregiver, teacher or coach. Traditionally, athlete performance preparation has been dominated by such externally driven organisation, with practitioners prescribing augmented information in the form of verbal instruction and continuous, sequential, corrective feedback directing athletes towards the reproduction of putative templates of performance behaviours [5].

An important direction of constraint on athlete self-regulation in performance concerns the exploitation of inherent self-organising tendencies for individuals to locally adapt and adjust to emerging competition demands, from an internally driven source. From an ecological ontology, ‘self-regulation’ refers to the development and exploitation of deeply intertwined, functional relationships between a performer’s actions, perceptions, intentions, emotions and the environment [6]. This interpretation differs from the orientation of self-regulation in cognitive psychology defined by Zimmerman [7], p. 14 as “…self-generated thoughts, feelings and actions that are planned and cyclically adapted to the attainment of personal goals”. An important challenge here has been to understand what the ecological conceptualisation of performance regulation in athletes and teams signifies for the practice of coaches and supporting scientists.

Over the years, applied scientists working in the theoretical framework of ecological dynamics, have re-conceptualised the role of practitioners in athlete development and performance preparation [8–10]. This re-conceptualisation advocates the notion of practitioners as *designers*: professionals who harness the continuous, non-linear and deeply integrated interactions emerging between the performer, task and environmental subsystems [11, 12]. Such a re-conceptualisation is user centred, placing the *athlete-environment interaction* at the core of the learning process, and views the coach as an integral member of a multidisciplinary team of support practitioners who co-design representative and information-rich practice environments [13, 14]. This multidisciplinary organisation has been framed as a Department of Methodology [14], which unifies practitioners and applied scientists with a common conceptualisation of performance and development, goals and language.

During the last two decades, research has provided theory and data for the establishment of ecological dynamics as an important theoretical framework for performance preparation in sport [15–21]. Here, performance preparation is viewed as context dependent, being a means of preparing performers (e.g. children or elite athletes) for immediate sporting involvement (e.g. acute engagement and enjoyment or preparation for an upcoming competition). Athlete development, on the other hand, can be seen to occur over the longer timescales (e.g. transiting from junior to senior competition, sustaining high-performance participation and prolonged success). Currently, targeted research is guiding the work of professionals in the practical integration of relevant propositions within specific sporting environments (for some notable examples, see [10, 13, 22–26]). Continued examples of implementing an ecological dynamics framework by sporting practitioners could support those who seek to avoid reverting to more traditional models of performance preparation grounded in ‘operational standards’ or ‘technical performance templates’ prescribed in coaching manuals. Accordingly, the aim of this article is to offer two case examples of its practice integration across the spectrum from high-performance to developmental sporting environments. Specifically, the following sections disclose the integration of ecological dynamics for performance preparation in (1) elite Australian football, guided by a concept referred to as ‘Heads Up Footy’; and (2) Swedish youth Association Football, guided by a concept referred to as ‘Football Interactions’. In these examples, our intention is to drive the continued methodological advancement of the application and integration of ecological dynamics in high-level sports.

## Case example 1

### Integrating the Head Ups Footy concept for performance preparation in elite Australian football

**Fig. 1:**
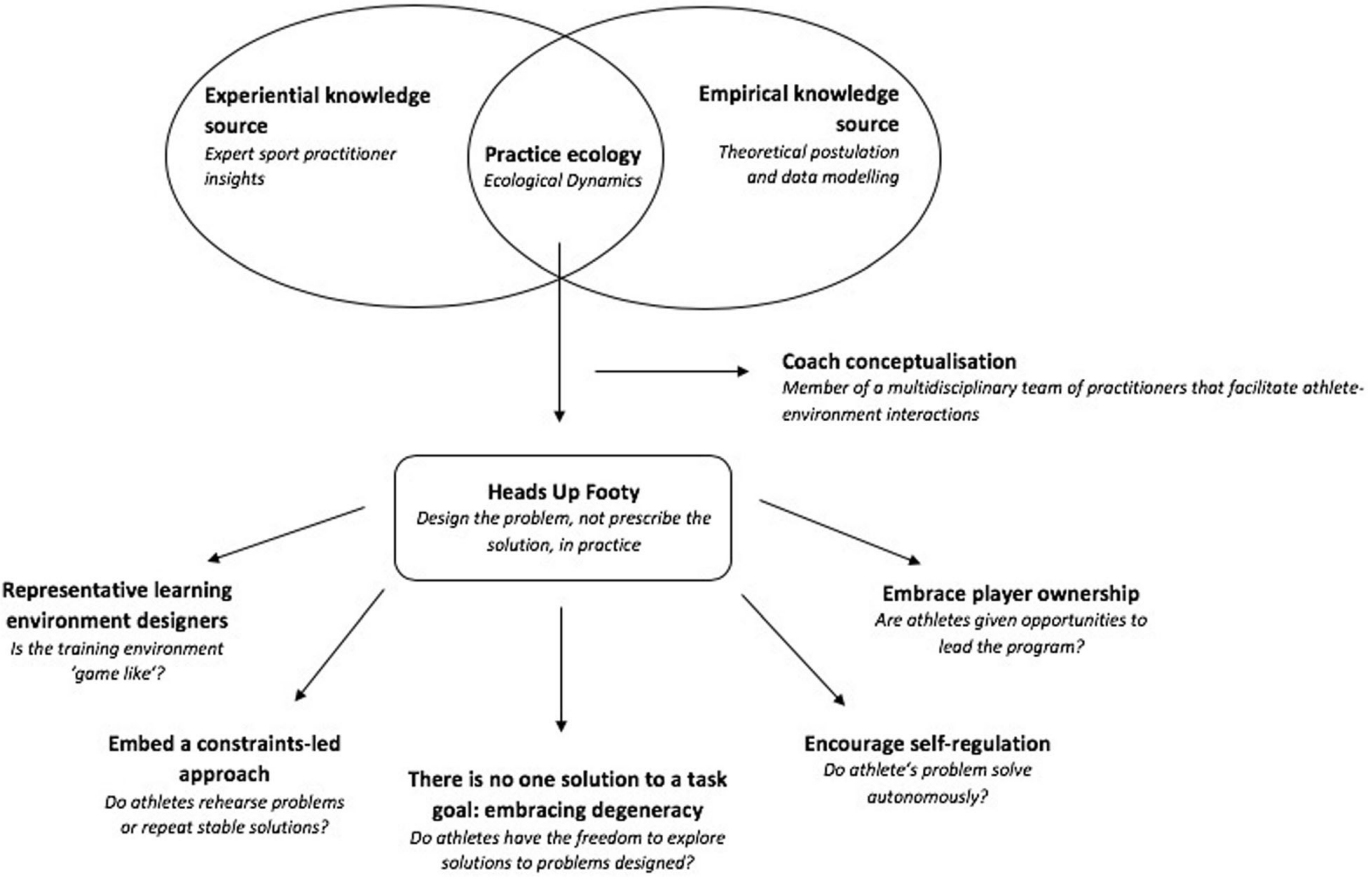
A conceptual overview of Heads Up Footy

The application of an ecological dynamics framework in sport is growing, yet challenging, with Renshaw and Chow [23] citing the ‘dense academic language’ typical of such frameworks as a global constraint on the work of practitioners wanting to understand applications of its key concepts. An important task for coach educators advocating the use of constraints in performance preparation is, therefore, to provide a user-friendly platform for practitioners interested in adopting such an applied scientific approach to their work [23]. In this case example, a guiding framework was developed for performance preparation in elite Australian football that supported interpretation and transference of key concepts to practitioners responsible for bringing practice to life. This framework was theoretically, empirically and experientially informed, and as such, in an attempt to capture the individual environment, self-regulating and adaptable foundations of ecological dynamics, whilst offering sporting practitioners meaningful and transferrable terminology, this framework was referred to as ‘Heads Up Footy’ (Fig. 1).

### Knowledge sources

The first design feature of this framework is the interaction between the knowledge sources, blending and exploiting existing experiential and empirical knowledge on ecological dynamics and application of its key principles. As highlighted elsewhere [27], sport science has focused on developing empirical support for performance preparation, pioneering the theoretical vibrancy of many areas. However, this has often been treated as the sole knowledge source that sport scientists need for designing practice environments, ignoring the experiential knowledge accrued by expert sports practitioners gained from years of experience working with athletes and teams in rich and varied landscapes. Experiential understanding should be treated as a rich knowledge source that, if used in a complementary way with empirical research, can guide the successful integration of performance preparation models in sport [24, 27, 28]. Others (e.g. [29]) have considered how sporting organisational cultures can facilitate co-operation between individuals, knowledge sharing, embedded interactions and sound operationalisation for the development of productive talent development environments. Thus, a critical tenet of the Heads Up Footy framework was to facilitate the interaction between empirical (data and theory on complex adaptive systems) and experiential knowledge to underpin the practice environment. By doing so, the practice ecology could preserve the fundamental conceptualisation of ecological dynamics (guiding empirical knowledge), whilst concurrently making the key concepts translatable for sporting practitioners, allowing them to draw on their experiential knowledge to create meaning specific to practice designs in Australian football.

### Coach conceptualisation

The next design feature was the re-positioning of the coaches’ role in performance preparation. As discussed by Woods et al. [10], when conceptualised through an ecological dynamics framework, the role of a coach evolves from a provider of verbal corrective instruction, to a learning environment *designer*, who facilitates athlete-environment interactions. In this role, re-conceptualisation, the coach is responsible for identifying and manipulating key constraints of the practice environment in an attempt to guide the attention of performers to regulatory information sources available in the surrounding landscape [3, 12]. An important feature of this approach is that the practice landscape can be co-designed *with* the athlete, placing their needs at the centre of the performance preparation model. Further, the re-conceptualisation of the coaches’ role in performance preparation requires an understanding that they are integral members of a multidisciplinary team of sporting practitioners that work together to design individualised learning environments [14]. This appreciation is critical, as it prevents performance dissonance amongst practitioners, which could lead to ‘siloing’ [30]: individual practitioners who work in isolation with performers focusing separately on physical, technical, psychological or tactical aspects of performance. Within this multidisciplinary team, it is imperative that the group of sporting practitioners share integrative tendencies that are based on both rich empirical and experiential knowledge sources [14]. This approach could subsequently facilitate the resolution of behaviours that are considered desirable for team and/or athlete success (product), in addition to identifying interacting constraints that shape behavioural emergence (process).

In the remaining sections of this paper, we unpack other important design features of this framework. Accompanying the empirical conceptualisation of each design feature is a hypothetical example applied to Australian football (experiential knowledge), allowing the reader insight into how such a concept could be brought to life in practice.

### Representative learning designers

By identifying critical sources of information that support utilisation of relevant affordances (defined as opportunities for action, see [31]), a coach can carefully design learning activities that *represent* or faithfully simulate competition demands. Founded on initial insights of Brunswik [32], and later work of Araújo and colleagues [17, 33, 34], this type of practice process is referred to as *representative learning design*. Representative training activities are high in specificity of information sampled from a competitive performance environment, which is to be designed into practice task settings. As shown by Pinder and colleagues [35, 36], representative learning design is predicated on the integration in practice and training programmes of relevant informational constraints experienced within particular competitive performance environments. Exposure to relevant task and information constraints helps athletes to learn to perceptually attune to relational affordances of a particular competitive landscape. It is this ongoing attunement (to information) that subsequently directs athletes and teams towards a deeply entangled and highly functional relationship with a competitive performance environment, referred to as their *ecological niche* [1]. This athlete-environment scale of analysis for explaining specificity of practice effects on skill acquisition differs from the internalised neuromotor impulse rationale proposed in early motor learning theories [1]. With this empirical understanding in mind, how could a coach design and subsequently monitor the representativeness of their learning designs?

#### Example 1 - Is the training environment ‘game like’?

An important feature of successful performance within Australian football is effective ball disposal between teammates, which can occur via a handball or kick. To design representative learning environments, a practice task needs to be guided by information sources that shape actions and behaviours within competition. Thus, informational constraints could be *sampled* from competition to allow them to be designed into a practice activity which simulates the competitive performance environment.

One strategy to facilitate the sampling of constraints could be to ask a coach to heuristically select key constraints they perceived to shape kicking actions. Through performance analysis, these constraints (such as ‘time in possession’ or ‘physical pressure on the ball carrier’) could then be sampled from competition and practice landscapes, allowing a coach to base his/her experiential knowledge on performance data from a database of relevant kicks performed in competition. For example, when the same notational analysis is applied to a practice task intended to augment kicking skill, a coach could contrast the sampled constraints from competition and the practice task (such as ‘time in possession’) to ensure that a specific training activity was more ‘game like’ or not. To visualise such an approach, a performance scientist could plot the percentage of total kicks performed within different temporal epochs (‘time in possession’ constraint split into < 2, 2-4 and > 4-s epochs, for example) from both competition and practice landscapes, enabling a concise identification of potential points of difference. These performance data could offer more detailed insights into determining where (if any) mismatches between training and competition environmental demands may exist, providing a basis for training activity re-design to more closely align the constraints observed during game play. By engaging performers to discuss their performance needs, this co-design approach can create more ‘game like’ training activities. Clearly, greater depth of, and diversity in key constraints and their interaction sampled from both competition and practice landscapes, would enable deeper insight into the representativeness of training tasks. One way to achieve this could be through the use of more advanced machine learning techniques, such as rule induction (for detailed methodological insight, see [25]).

### Embedding a constraints-led approach

A fundamental implication of ecological dynamics is the rationale that the concept of skill acquisition could integrate the notion of ‘skill adaptation’ (for detailed arguments see [18]), being defined through the development (acquisition) of a highly functional and evolving relationship between an athlete and a competitive performance environment. Such a perspective on skill performance was initially proposed by Bernstein [37] in the notion of dexterity, defined as the “*the ability to find a motor solution for any external situation, that is, to adequately solve any emerging motor problem correctly* (i.e. adequately and accurately), *quickly* (with respect to both decision-making and achieving a correct result), *rationally* (i.e. expediently and economically) *and resourcefully* (i.e. quick-wittedly and initiatively)” (italics in the original) (p. 134). In contrast to early connotations of specificity of practice, Bernstein’s [38] insights clarified that the demand for dexterity was not in the movements themselves, but in a performer’s adaptability to the surrounding environment.

The implications of this ecological conceptualisation of ‘skill’ are important to consider for sporting practitioners, as it suggests that practice tasks should promote an environment in which athletes are faced with continual problems, which they are required to solve. To enable this design approach, and aid ensuing exploration, a team of practitioners could consider the manipulation of a range of key constraints to educate an athlete’s attention towards features of their environment critical to the solving of emergent problems specific to his/her action capabilities. A guiding framework to assist with the manipulation of constraints is that proposed by Newell [11]. The key question is: how could practitioners manipulate practice task constraints to guide perceptual attunement and encourage adaptable performance solutions to emergent problems experienced in competition?

#### Example 2 – Do athletes rehearse problems or repeat stable solutions?

Questions such as: *do athletes rehearse problems or repeat stable solutions?*, could capture the fundamentality of a constraints-led approach (guiding perceptual attunement and encouraging athlete adaptability), whilst affording a digestible platform for practitioners responsible for bringing it to life via their experiential knowledge. In this following example, a practice task consisting of a constraint manipulation is discussed with reference to the promotion of perceptual attunement and adaptable performance solutions to an emergent tactical problem.

Match simulations are a common training task within performance preparation frameworks in elite Australian football environments. To guide the perceptual attunement of players within these simulations towards the solving of dynamic, emergent tactical problems, a coach could consider artificially manipulating practice game scorelines. Specifically, by strategically placing one team marginally in front (and one marginally behind) towards the end of the match simulation, a coach could encourage self-organised player-environment interactions, as both teams search their performance landscapes for affordances that allow them to either preserve or (re)gain the lead.

To quantify emergent ball passing interactions between the players, following the constraint manipulation (defined here through the tactical problem), performance analysis could be used in conjunction with principles of the constraints-led framework discussed earlier. Specifically, constraints shaping kicking between teammates could be sampled “pre- tactical problem” (i.e. before a score-imposed change) and “post-tactical problem” (i.e. after a score-imposed change). The distribution of kicks within a certain constraint category could then be compared between conditions to facilitate insight into possible ball passing interactions in response to the tactical problem. This would ultimately furnish the coach insights into how the players self-regulate performance in an adaptive response to constraint manipulation. This process assists the coach in identifying the informational constraints that players detect when attempting to solve emergent problems within competition, thus enabling them to manipulate these features to educate a player’s attention in future practice designs. As per the first example, understanding passing interactions could be further enhanced through the utilisation of more advanced analytical techniques, such as network analysis [4]. Such analyses would enable deeper inferences into the collective behaviours of players at a local-to-global scale of analysis in response to an environmental constraint [4].

### There is no one solution to a task goal: embracing degeneracy

A central tenet of ecological dynamics is the appreciation of an athlete or team as a *complex adaptive system*, in which the non-linearity and dynamics of performer-environment interactions continually invite actions and behaviours towards the achievement of the same, or similar, task goals [39]. Accordingly, performance solutions to an emergent task goal are highly nuanced to the environment and action capabilities of the performer. This characteristic, within ecological dynamics, has been conceptualised through the notion of *system degeneracy*, a concept that describes how the same system output can emerge through the use of structurally different elements or configurations [40].

Given the re-positioning of skill acquisition as ‘skill adaptation’ within ecological dynamics, it is the progressive attunement to relevant continuously emerging and decaying affordances that a coach should consider within their practice designs, not the rehearsal of the same (static) solution to the task goal. It is through this attunement process that an athlete can learn to functionally adapt movements to exploit key constraints to achieve the same task goal [41]. Thus, practice designs should expose athletes to the general ecology of a performance landscape, enriching their skills base so that they can exploit multiple opportunities for action that emerge in competition [18]. For this reason, learners need a nuanced balance between generality and specificity of practice (expressed in terms of informational constraints and problems/challenges faced) [1]. For example, at the specialised end of this practice continuum, there would be fewer, but more specific, affordances relating to the achievement of a specific task goal. Comparatively, towards the other more generalised end of this continuum, there would be a more diverse and extensive range of affordances relating to more global and less specific task goals. Put more directly, athletes need to be free to explore different and varied regions of their performance landscape in the achievement of task goals, with the challenge for practitioners being to know when to inhabit such regions within their practice designs.

#### Example 3 - Do athletes have the freedom to explore solutions to problems designed?

In recognition of the empirical knowledge on system degeneracy, and in a similar vein to the design features previously unpacked, questions such as: *do athletes have the freedom to explore solutions to problems designed?*, draws the attention of sport practitioners to inherent degeneracy tendencies described in the following example. In this practice design, two teams are tasked to deceive opponents to either maintain or obtain ball possession by any means they felt necessary to achieve this task goal. To promote these functional behaviours, a coach could first anchor points or a score to successful deceptive actions, immediately channelling the player’s attention towards the utilisation of deceptive affordances offered within the performance landscape. Second, to promote self-regulated exploration of a variety of deceptive behaviours, a coach could use team convolution, exemplified through the environmental constraint manipulation of placing competing teams in the same coloured bibs during practice games. Such a constraint manipulation would increase practice task difficulty by challenging players to self-regulate by using scanning behaviours to search for, discover and explore affordances for passing the ball offered in the revised performance landscape.

To observe emergent deceptive behaviours, a coach could then quantify the type of deception strategy actualised by the players within the practice task. Designing a practice landscape that facilitates manipulation of constraints for task goal achievement will challenge players to search for multiple opportunities for action, and not rehearse one (static) performance solution. Task goals could be achieved by exploiting the use of structurally different system elements (intertwining cognitions, perception and action in performance).

### Encourage self-regulation

Conceptualised through ecological dynamics, self-regulation broadly emphasises emergent interactions between a performer and the environment. From this perspective, performers learn to self-regulate through the acquisition and exploitation of functional relationships between their actions, perceptions, intentions, emotions and environment [6]. Exposure to rich and varied practice environments promotes opportunities for performers to develop *knowledge of* [31] their performance environments that they can learn to self-regulate and adapt stable perception-action couplings to emergent problems encountered within competition. A key challenge for coaches is understanding how to create conditions within practice landscapes that afford opportunities for athletes to continuously self-regulate their coupling of perception and action.

#### Example 4 - Do athlete’s problem solve autonomously?

To capture the fundamentality of self-regulation conceptualised through ecological dynamics, questions such as *do athlete’s problem solve autonomously?*, could be commonly raised amongst a team of practitioners. To facilitate this process within practice designs and assist players in their capability to self-regulate their perception-action couplings without global intervention from a coach, questioning could be an effective strategy [42]. Questioning affords the coach with the opportunity to channel the attention of players to critical information sources within their practice and performance landscapes that may assist them in the solving of an emergent tactical problem. However, the important feature of such a strategy to promote self-regulation is that questioning from an ecological dynamics perspective does not involve the player verbalising their reasoning and structured response (capturing the notion of *knowledge about* the environment, [31]). Rather, the aim of questioning through ecological dynamics is to direct the player’s attention towards a relevant field of affordances to be actualised such that they can respond with *knowledge of* the performance environment [31], exemplified through actions, perceptions and skilled intentionality [1]. Some examples of questioning to promote self-regulation being actualised may include (but are not de-limited to) the following:

1. Questioning that draws player attention towards number inequalities (overloads or underloads) in certain field locations.
  *Knowledge of* these number inequalities could subsequently lead to the self-organised exploitation of functional movement strategies, facilitated by scanning with and without the ball, when outnumbering or being outnumbered by opposition.
2. Questioning that draws player attention towards environmental features likely to influence ball disposal (such as effects of wind, rain or extreme heat).
  *Knowledge of* these extrinsic environmental features could lead to self-organised ball disposal interactions between teammates, such as resting with the ball in extreme heat to preserve anaerobic capacity.
3. Questioning that draws player attention towards tactical strategies imposed by an opposing team (for an example in volleyball over a whole season, see [43]).

### Embrace player ownership

The last feature of Heads Up Footy is the appreciation of a learner-centred environment, allowing individual needs to be prioritised within practice designs [9]. As discussed throughout this article, such an appreciation has implications for the coach’s role in performance preparation, who works with the athlete to co-design landscapes representative of competition [10]. This co-design process places each athlete’s needs at the core of the development and performance preparation process. Through association, athletes gain a greater opportunity to engage with the learning environment. So, how does a coach place an athlete at the core of the learning design and promote opportunities for players to take ownership of their learning process?

#### Example 5 - Are athletes given opportunities to lead the programme?

As in other design features, a multidisciplinary team of practitioners could use questions such as *are athletes given opportunities to lead the programme?*, to support player engagement and autonomy. Such an approach can bring to life the often-misunderstood concept of athlete-environment-centred, widening understanding of what constitutes ‘experiential knowledge’ in high-performance sport. It affords athletes’ input on integral parts of their learning environment, focusing their attention on the relative value of their experiential knowledge from years of competitive performance. To facilitate this process, and afford opportunities for players to lead their performance development programme, a few strategies are described below:

1. Embrace the notion of co-design within practice tasks
  *Example*: Including players (where possible/appropriate) in discussions orienting the specific design of practice tasks. This approach enables deeper insights into what affordances players perceive and actualise within their landscapes (which coaches can only understand from a second-hand perspective), allowing the design of tasks that better represent competition demands, in addition to informed constraint manipulation to educate attention.
2. Management of time within weekly schedules
  *Example*: Players being free to manage aspects of their preparation perceived to need additional support. This could include (but is not delimited to) additional education, recovery strategising and/or additional work on specific skill, mental and physical condition and tactical development.
3. Facilitate player-led training sessions
  *Example*: Allowing players opportunities to autonomously (without continuous coach interaction/input) design, implement and review training activities. By doing so, it is likely they will develop richer *knowledge of* their environment through the design and reflection of practice tasks that invite, guide and regulate the actions and behaviours of teammates.

## Case example 2

### Re-conceptualising player development in youth football: the ‘Football Interactions’ concept

The ‘Football Interactions’ concept emerged from an ecological realism perspective, with talent development practices not being based on deterministic models of behaviour (e.g. focused on action rehearsal or reproduction), but founded upon high-quality athletic experiences and continuous interactions with practice and competitive environments. Accordingly, in April 2017, with the support of a newly-formed Research and Development department comprised of researchers and coaches, AIK (Allmänna Idrottsklubben) youth football made the decision to build a player development framework guided by (i) the well-being of the child; (ii) supporting documents from the United Nations Convention on the Rights of the Child and Swedish Sports Confederation, and (iii) the promotion of more youth players to participate in the under 16, under 17 and under 19 years teams. After implementation, this approach saw the disbanding of AIK’s traditional early talent selection policy, in which the club had selected the ‘best’ early performers to form an academy team at < 9 years of age.

Whilst coined by the Research and Development department, the Football Interactions concept was predicated on Wittgenstein’s [44] notion of *form of life*, that acknowledged the many values, beliefs and different socio-cultural practices (e.g. in practice task design and coach education) that shaped player development, and especially, Gibson’s [31] and Rietveld and Kiverstein’s [45] accounts of affordances. An in-house investigation into the form of life at AIK youth football using ethnographical strategies was then carried out to inform present and future possibilities of evolving practice and player development [27]. Specifically, a contribution of observations, field notes, document analysis and unstructured interviews led to the resolution of areas of refinement with regards to the practice and learning environments currently designed at AIK youth football. The following section summarises some of the outcomes of these ethnographic strategies, uncovering key areas that required attention for the organisation to realign practice within an ecological dynamics framework.

### Recognising a *form of life* based on actions and a culturally pervasive planning heritage

Integrating an ecological dynamics framework for player development in a youth football club can be a challenging task, which can be compounded by a *path dependency* underpinned by inherited beliefs sheltered by more traditional ideological inertia [46]. In this context, path dependency refers to a practitioner’s reliance on prior experiences or beliefs to inform the integration of current practice. For example, a traditional feature of Swedish coach education programmes and talent identification initiatives orient coach centred and early identification practices, two concepts with limited scientific support [46–48]. Accordingly, although blending experiential and empirical knowledge sources was an integral component of the Football Interactions concept, it was first acknowledged that there could be convolution between experiential knowledge gained through rich and varied experiences, and experiential knowledge simply gained through the passage of time. The latter of these two experiential knowledge sources could incur stagnated path dependency (i.e., practice based on some form of sheltered and traditional ideology), if the practitioner was simply exposed to the same ecology over some prolonged periods of time. Differentiating the types of experiential and empirical knowledge to be drawn upon for implementation was an essential feature of the Football Interactions concept.

Through biographical examination, it was identified that coaching skill was being developed and shaped by the landscape of traditional coaching practices and coach education programmes, with these being recognised as key constraints on the emergence of new, more contemporary epistemologies. A further revelation was how attributes and skills appreciated in players at AIK youth football were culturally embedded in traditional pedagogical approaches, organisational settings and structural mechanisms founded upon specific socio-cultural and historical constraints. For instance, training designs in Swedish youth football have typically been underpinned by a culturally dominant planning paradigm pervasive in traditional educational approaches (e.g. coach determines in advance the specific theme, presents predetermined coaching points and controls the sequence and duration for each part of the session). Within the younger teams at AIK youth football, it was revealed that coaches’ planning and practice designs were aimed at shaping self-organising tendencies of players and teams at a global-to-local scale by explicitly imposing a game model [4]. Put simply, youth players were seemingly ‘props’ in some type of coach-conducted orchestration, where players learned to play an idealised model of the game as opposed to functioning in the game itself, limiting player autonomy and self-regulating tendencies. To try to control future outcomes, the actions of young players were routinely ‘drilled’ in choreographed practices to perform predetermined passing patterns to be later regurgitated in competitive games. So, to provide insight as to why certain coaching practices enhanced or diminished outcomes, there was a need to help coaches recognise the impact of their interventions by understanding what is contextually more (in)appropriate or (un)functional. It was recognised by the AIK Research and Development department that part of the re-conceptualisation process at the level of practice task design required the liberation of the coach from the dominant historical and cultural ideas and tendencies.

### Evolving towards a *form of life* based on Football Interactions

To initiate this liberation, the framework ‘AIK Base’ was introduced by AIK Research and Development in late 2018 (Table 1), containing a collection of concepts and references that formed a foundation for practice design and education programmes. Global-to-local processes, amplified in a coaching culture where team organisation and the notion of a putative ‘optimal’ technique, had previously been prioritised over developing players’ understanding ‘in’ the game. As this had an over-constraining influence on players’ local interactions, it was proposed that by adopting these references within the AIK Base, coaches could help young players learn how to co-adapt to the performance environment using local information sources in order to harness local-to-global tendencies for self-organisation (see [49].) Grounded in the theoretical framework of ecological dynamics, coaches at AIK were encouraged to adopt principles of a constraints-led approach to skill learning [23, 50]. This approach included the use of informational constraints related to questioning [1], which as described in the first case example, guided the attention of the players towards important features of the environment in solving performance-related problems. They were not intended to be answered by the players with verbal responses, typified in more traditional sporting pedagogies, but were implemented to guide the players towards the actualisation and utilisation of relevant and soliciting affordances within the environment [1]. The notion of Football Interactions was, therefore, introduced to shift the coaching narrative away from implementing predetermined ‘optimal’ techniques or patterns, towards developing a more adaptive, interactive performer, guided by emerging information and affordances of the performance environment. Further, football was defined as a dynamic team sport, in which players routinely switched between attack and defence phases of play. This dynamic offensive and defensive flux, underpinned by the ecological dynamics framework and led by a modified three-stage learning model (search and exploration; discovery and stabilisation; exploitation (see [51])), informed ‘principles of play’ at AIK youth football.

**Table 1:** The theoretical framework underpinning the AIK Base

Theoretical framework	Ecological dynamics: constraints led approach
**Pedagogical concept**	**Nonlinear pedagogy**—e.g. (i) Representative learning design, (ii) repetition without repetition (adaptive movement variability), (iii) manipulation of constraints, and (iv), designing opportunities or affordances for developing relevant information-movement couplings.
**Football concept**	**Football Interactions (pass, dribble, off-ball movement)—**refers to how a player coordinates his/her behaviour within the performance landscape in relation to that environment, on the basis of, not only the immediate physical and informational (i.e., situational) demands but also underpinned by socio-historical and cultural factors*.*
**Principles of play**	**In possession:** Search, discover, exploit space and gaps using football interactions. **Recovering the ball:** Close space/gaps, minimise possibilities for opponent’s football interactions, win the ball.

### Designing practice tasks that promote Football Interactions

Emerging behaviours revealed in football interactions can be observed and facilitated through carefully designed practice tasks informed by ‘principles of play’ rather than a rigid scheme of behaviour (typified in ‘game models’). Football interactions are tuned by environmental information to function specifically in each unique situation, emphasising the need to understand the nature of the information that constrains movement. In stark contrast to predetermined passing patterns, practice should highlight informational constraints that allow players to learn new ways of acting adaptively through exploration [52]. The practical implication of this approach is that, instead of rehearsing one solution, players should be invited to search their affordance landscape to improve the coupling of perception and action and promote the actualisation of relevant affordances through football interactions. Two applied examples of football interactions being actualised within practice design are described below.

#### Example 1 - Designing a practice task based on Football Interactions to invite opportunities to ‘dribble’

A central component of football performance is being able to ‘dribble’ the ball (that is, to maintain ball possession whilst running). Thus, performance preparation within developmental programmes framed by ecological dynamics should educate players of opportunities to dribble that may emerge, as opposed to the repetition of the ‘football action’ (dribbling) itself. This example draws upon a 4v4 game, in which affordances orienting start positions were designed in to initially educate the player’s attention towards relevant information sources to exploit gaps and utilise space whilst in possession of the ball. To further promote the utilisation of gaps and space via dribbling, as opposed to passing, a coach could manipulate the task in such a way that promotes the utility of dribbling. To do so, careful task constraint manipulation could be used, such as awarding a point to the team who is able to intercept a pass, thus placing a risk associated with passing the ball, but not excluding its utility. This increased risk could invite players both with and without the ball to self-organise their individual and collective behaviours by attending to local information through utilisation of football interactions (which, in this case, orients passing, dribbling and off the ball movement to support the player in possession). Whilst the targeted task constraint manipulation to increase risk or uncertainty associated with passing emphasises the need to identify opportunities to exploit gaps and space through dribbling, it additionally invites teammates to continuously adapt their position in relation to local information (e.g. teammate in possession, and positioning of nearest opponents). This example yields stark contrast to more traditional ways of ‘teaching’ dribbling, which would typically involve the reproduction of predetermined dribbling patterns.

#### Example 2 - Co-designing practice tasks to facilitate goal shooting

A key aim of the Football Interaction concept was that the affordance landscape was to be co-designed between the coach and player(s). In other words, practice tasks were co-designed between players (through intentions revealed in their football interactions and reflections) and coaches (through observation of these interactions and reflection). Through co-design, coaches could become better informed with regards to designing in present and future opportunities or affordances for interaction [53]. In this example, an affordance landscape was co-designed between players and coaches when practicing goal shooting.

It is quite common in youth football to see shooting exercises in which the coach drives the action of the player, as opposed to exercises in which the football interaction is preserved (such as shooting in relation to situational information). Thus, to co-design a shooting practice task that places the football interaction at its core, a coach could observe how the player is adapting his/her shooting behaviour in relation to the information present (such as positioning of the goalkeeper, who primarily invites the shooting affordance). Through this observation, and subsequent player reflection, a coach could better understand the information sources players use to guide their shooting behaviour, being able to design in these information sources to promote richer football interactions through careful constraint manipulation (such as making the goal width larger or smaller to accentuate goalkeeper movements, thus inviting opportunities for gap exploitation through educating the attention of the shooter). This is in direct contrast to traditionally focusing on *how* the player is performing the shooting action.

In summary, this case example sought to offer readers a basis of how practitioners could integrate key features of ecological dynamics in the development of youth footballers. Specifically, it emphasised the evolution of more historical coaching practice, with practitioners transitioning towards learning environment designers that placed the individual-environment (football) interaction at the core of the learning design.

## General Conclusions

As timelessly captured by the psychologist Kurt Lewin, a good theory should be practical. Thus, an important current and future challenge for the theory of ecological dynamics resides within its practical integration. We sought to provide insights into how high-level organisations have attempted to integrate ecological dynamics for performance preparation. It was not our intention to prescribe a universal solution for performance preparation, but rather offer the readership an overview on how some professional sporting organisations are seeking to challenge traditional ideologies of performance preparation. More specifically, these case examples were intended as models exemplifying how practitioners and organisations could challenge themselves to adapt strategies to design contemporary practice tasks within their ecosystem. To continually assist this process, we encourage the sport science community to promote the sharing and scientific publication of exemplars and/or case studies that afford opportunities to accept, reject or adapt practical approaches used by others. We perceive that it is this continued sharing, offering and discussion of application and methodological ideas in the sport sciences that will advance the application of (good) theory.

## Data Availability

Not applicable.
